# Oleic Acid and Insulin as Key Characteristics of T2D Promote Colorectal Cancer Deterioration in Xenograft Mice Revealed by Functional Metabolomics

**DOI:** 10.3389/fonc.2021.685059

**Published:** 2021-08-09

**Authors:** Ying Zhang, Di Wang, Bo Lv, Xiaoying Hou, Qiwei Liu, Chuyao Liao, Ruijie Xu, Yuxin Zhang, Fengguo Xu, Pei Zhang

**Affiliations:** ^1^Key Laboratory of Drug Quality Control and Pharmacovigilance (Ministry of Education), State Key Laboratory of Natural Medicine, China Pharmaceutical University, Nanjing, China; ^2^Nanjing Drum Tower Hospital, The Affiliated Hospital of Nanjing University Medical School, Nanjing, China

**Keywords:** insulin, oleic acid, colorectal cancer, synergistic effect, functional metabolomics

## Abstract

Colorectal cancer (CRC) is one of the most commonly diagnosed cancers with high mortality worldwide. Type 2 diabetes mellitus (T2D), known as a risk factor of CRC, can promote the deterioration of CRC, but the underlying mechanism is elusive. In this study, we aimed to reveal the relationship between CRC and T2D from the perspective of small-molecule metabolism. First, a list of common dysregulated metabolites in CRC and T2D was obtained by retrieving existing metabolomics publications. Among these metabolites, oleic acid (OA) was found to be able to promote the proliferation and migration of colon carcinoma cell HCT116. Further experiments proved that insulin could significantly strengthen this promotion and showed a synergistic effect with OA. Mechanism study found that OA and insulin acted synergistically through the extracellular signal-regulated kinase (ERK)1/2/c-Myc/cyclin D1 pathway. In addition, the combination of ERK1/2 inhibitor SCH772984 and cyclin-dependent kinase (CDK)4/6 inhibitor palbociclib showed a remarkable inhibitory effect on tumor growth *in vivo*. Taken together, the current study found that OA plays an important role in CRC development by using a functional metabolomics approach. More importantly, insulin and OA were confirmed to synergistically promote the deterioration of CRC *in vitro* and *in vivo via* ERK1/2/c-Myc/cyclin D1 pathway. Our findings may shed light on CRC treatment among the T2D population.

## Introduction

Colorectal cancer (CRC), one of the most common malignancies, has a high incidence and mortality rate globally. In 2020, it is estimated that 1.93 million people were diagnosed with CRC, and >900,000 died from CRC worldwide (1). While the 5-year survival rate after curative surgery reaches ~90% at the early stage of CRC, it decreases to only 14% when distant metastasis occurs (2). The major risk factors of CRC are age and family history (3); however, studies have shown that CRC is also highly associated with unhealthy lifestyles like alcohol consumption and smoking (4) and diseases such as obesity (5), inflammatory bowel disease (6), and diabetes (7). Particularly, diabetes is a risk factor for multiple cancers such as pancreatic ductal adenocarcinoma (8), hepatocellular carcinoma (9), colon cancer (10), and breast cancer (11). The International Diabetes Federation (IDF) reports that the number of diabetes patients would increase from 415 million in 2015 to 642 million by 2040 (12). It has been discovered that the incidence of CRC in the type 2 diabetes mellitus (T2D) population increases up to three times compared to that of the general population (12). Also, diabetes is associated with a reduced survival rate and increased relapse risk in CRC patients (13).

It has been reported that type 1 diabetes (T1D) is associated with only 7% of the increase in cancer risk, while T2D has a greater impact, indicating that the single chronic hyperglycemia environment in diabetes patients is insufficient to aggravate cancer deterioration (14). Hormonal (insulin resistance), inflammatory, and metabolic characteristics of diabetes are potential links between diabetes and CRC (15). Moreover, studies found that diabetogenic levels of glucose and insulin can regulate the growth and migration of colon cancer cells (16), which may be helpful for understanding the relationship between T2D and CRC. Signal pathways such as AMP-activated protein kinase (AMPK), mammalian target of rapamycin (mTOR), and sirtuin 1 (SIRT1) and autophagy-related pathways may also bridge T2D and CRC and participate in cancer progression (12). MEK1/2/ERK1/2/c-Myc/cyclin D1 is an important pathway in the development of CRC, which may take part in T2D-associated CRC deterioration progress as well. In addition, as the terminal of biological process *in vivo*, metabolites are associated with both T2D and CRC development and may serve as a bridge linking these two diseases. It has been reported that the level of metabolites such as octanoic acid, decanoic acid, and histidine are correlated with CRC stages (17), and high plasma levels of leucine, valine, and branched chain amino acids (BCAAs) are inversely associated with colorectal adenoma (18). Endogenous metabolites such as BCAA and aromatic amino acids are significantly associated with the incidence of T2D (19). However, to reveal the metabolic connection of the two diseases, systematic retrieving and analysis of existing data generated from various metabolomics studies are necessary.

In this study, human metabolomics studies on CRC and T2D from 2000 to 2018 were retrieved. Common dysregulated metabolites found in both diseases were studied. Eventually, oleic acid (OA), a fatty acid upregulated in both CRC and T2D, was found to be able to promote the development of CRC. We further investigated the role of insulin and OA in the deterioration of CRC *in vitro* and *in vivo*. We discovered that in the presence of high insulin, OA could significantly aggravate the malignant phenotype of CRC *via* the ERK1/2/c-Myc/cyclin D1 pathway. These results provide a possible explanation for T2D promoting CRC development from the small-molecule point of view.

## Materials and Methods

### Materials and Reagents

Fatty acid-free bovine serum albumin (BSA) and recombinant human insulin were purchased from Solarbio Life Science (Beijing, China). Chemical compounds including leucine, isoleucine, valine, alanine, phenylalanine, serine, threonine, glutamic acid, glutamine, arginine, glycine, ornithine, tyrosine, proline, lysine, 3-hydroxybutyric acid (BHB), palmitic acid (PA), linoleic acid (LOA), stearic acid (SA) were purchased from Sigma-Aldrich (St. Louis, MO, USA). U0126-EtOH was purchased from MedChemExpress (Monmouth Junction, NJ, USA). The antibodies of ERK1/2, phosphor-ERK1/2 (Thr202/Tyr204) were purchased from Affinity Biosciences (USA) and c-Myc, cyclin D1, and alpha-tubulin were purchased from Proteintech (Chicago, IL, USA). Palbociclib and SCH772984 were purchased from Jiangsu Aikang Biomedicine Research Co., Ltd. (Nanjing, China). Isophane Protamine Biosynthetic Human Insulin Injection (Pre-mixed 30R) was purchased from Novo Nordisk (China) Pharmaceuticals Co., Ltd.

### Discovery of Common Dysregulated Metabolites in Colorectal Cancer and Type 2 Diabetes Mellitus

Human metabolomics studies on CRC and T2D from the year 2000 to 2018 were retrieved by using “Type 2 diabetes,” “diabetes,” “colorectal cancer,” “colon cancer,” and “metabolomics” as keywords in Web of Science (TS = “type 2 diabetes” OR TS = “T2D” OR TS = “colorectal cancer” OR TS = “colon cancer” OR TS = “CRC” AND TS = “metabolomics” OR TS = “metabonomics”) and PubMed (“type 2 diabetes” [All Fields]) OR (“T2D” [All Fields]) OR (“colorectal cancer” [All Fields]) OR (“colon cancer” [All Fields]) OR (“CRC” [All Fields]) AND (“metabolomics” [All Fields]) OR (“metabonomics” [All Fields]). As a result, 87 CRC-related literature and 72 T2D-related literature were obtained. The up- or down-regulated metabolites presented in these studies were extracted to generate a database. Dysregulated metabolites were listed, and those found in both CRC and T2D were highlighted for further verification. Functional metabolomics experiments were then designed to check whether these metabolites were able to affect CRC cell development.

### Cell Line and Cell Culture

Human colon carcinoma cell line HCT116 was purchased from Nanjing Hongxin Biotechnology Co., Ltd. (Nanjing, China) in 2019. HCT116 was cultured in high-glucose Dulbecco’s modified Eagle’s medium (DMEM) (Gibco, Grand Island, NY, USA) supplemented with 10% fetal bovine serum (FBS) (Gibco, Grand Island, NY, USA) and 1% penicillin–streptomycin in a humidified atmosphere containing 5% CO_2_ at 37°C. Cells were identified by Shanghai Biowing Biotechnology Co., Ltd. (Shanghai, China) using STR profiling in the year 2019. All cell experiments were performed with mycoplasma-free cells.

### Cell Stimulation and Oleic Acid–Bovine Serum Albumin Complex Preparation

After being washed twice with PBS, the cells were treated with 50 nM insulin in a serum-free medium for 6 h. Then, the cells were cultured with OA–BSA complex for 48 h. OA–BSA complex solution was prepared by stirring OA sodium salt at 55°C with 10% fatty acid-free BSA as Cousin et al. (20) described. The corresponding concentration of BSA was used as a control. Insulin was dissolved in 0.1 M HCl, and 0.1 M HCl was therefore set as control. In brief, a 100-mM fatty acid stock solution was prepared in 0.1 M NaOH by heating at 70°C, and a 10% (w/v) fatty acid-free BSA solution was prepared in ddH_2_O at 55°C. A 5-mM OA–10% BSA stock solution was prepared by adding 50 μl of the 100-mM OA solution dropwise to 950 μl of 10% BSA solution at 55°C. The mixture was cooled to room temperature before use.

### Cell Viability Assay

Cells were seeded in 96-well plates at a density of 1.5 × 10^3^ cells/well. After 24 h, cells were treated with Leu, Ile, Val, Ala, Phe, Ser, Thr, Glu, Gln, Arg, Gly, Orn, Tyr, Pro, Lys, BHB, lactic acid, pyroglutamic acid, OA, PA, LOA, SA for 48 h. Then, to investigate the synergistic effect of insulin and the above metabolites, cells were incubated in 50 nM insulin for 6 h before the metabolite stimulation. All of the treatments were in serum-free condition. After the stimulation (48 h), the cell viability was measured using an 3-[4,5-dimethylthiazol-2-yl]-2,5 diphenyl tetrazolium bromide (MTT) assay.

### Wound Healing Assay

Cells were seeded in 24-well plates at a density of 2 × 10^5^ cells/well. Confluent monolayer cells were scratched using a 20-μl pipette tip, washed with PBS three times, then added with fresh medium spiked with insulin and/or OA. Photographs were taken at 0, 24, and 48 h at the same location of the scratch with an inverted microscope (Nikon TS100, Japan). The wound area was analyzed by ImageJ software (NIH, Bethesda, MD, USA). The relative migration rate was calculated by the following formula: Relative migration rate (%, of control) = 100% × (Area _model, 0 h_ – Area _model, 24 or 48 h_)/(Area _control, 0 h_ – Area _control, 24 or 48 h_).

### Western Blotting

Cells were seeded in six-well plates at a density of 7 × 10^5^ cells/well in a complete medium. After 24 h, cells were treated with insulin 50 nM (INS), 200 or 500 μM OA in serum-free condition for 48 h. After being washed twice with phosphate buffered saline (PBS), cells were harvested in 1% phenylmethylsulfonyl fluoride (PMSF) radioimmunoprecipitation assay (RIPA) lysis buffer (Beyotime Biotechnology, Shanghai, China) and centrifuged at 14,000 rpm for 20 min at 4°C to collect the supernatants. The lysates were supplemented with sample loading buffer (Beyotime Biotechnology, Shanghai, China) and denatured at 100°C for 10 min. The protein content was measured by a bicinchoninic acid (BCA) protein assay kit (Beyotime Biotechnology, Shanghai, China). Then, the samples were separated on an 8% or 10% sodium dodecyl sulfate-polyacrylamide gel electrophoresis (SDS-PAGE) gel and transferred to polyvinylidene fluoride (PVDF) membranes. The membranes were blocked with 5% BSA in PBS containing 0.1% Tween 20 (PBST) for 2 h at room temperature, then incubated with primary antibodies overnight at 4°C. After being washed three times with PBST, the membranes were incubated with a secondary antibody conjugated to horseradish peroxidase (HRP; Proteintech) for 1.5 h at room temperature. Finally, the immunoreactive bands were visualized with an enhanced chemiluminescence (ECL) system on a Tanon 5200 chemiluminescent imaging system (Tanon Science & Technology). Densitometric analysis was performed using ImageJ software (NIH, Bethesda, MD, USA).

### Colony Formation Assay

Cells were seeded in six-well plates at a density of 1 × 10^4^ cells/well with a medium containing 10% FBS. Then, the cells were treated with insulin and/or OA for 2 weeks as described before. Colonies were fixed with 4% paraformaldehyde for 20 min and stained with 0.1% crystal violet solution for 10 min. Photographs were taken at the end of the experiment.

### Cell Apoptosis Assay

Cells were planted in 12-well plates at a density of 3 × 10^5^ cells/well. The cells were treated with insulin and OA as described before. Then, the cells were collected and washed twice with PBS and resuspended in 500 μl of binding buffer. The reaction between Annexin V-fluorescein isothiocyanate (FITC) and propidium iodide (PI) was induced at room temperature in the dark for 5–15 min. The results were analyzed immediately by a BD Accuri™ C6 flow cytometer (BD Biosciences, USA). Each experiment was independently performed at least three times. All the data were analyzed using FlowJo software (TreeStar, Ashland, OR, USA).

### Cell Cycle Assay

Cells were seeded in 12-well plates at a density of 3 × 10^5^ cells/well. The cells were treated with insulin and OA as described before. After 48 h, cells were harvested and washed with PBS. Then, 1 ml of DNA staining solution and 10 μl of permeabilization solution were added and vortex-mixed for 5–10 s and incubated in the dark for 30 min at room temperature. Finally, the cells were analyzed using a BD Accuri™ C6 flow cytometer (BD Biosciences, USA). All the data were analyzed by FlowJo software (TreeStar, Ashland, OR, USA).

### siRNA Infections

The target sequences of CCND1 were 5’-CCA CAG AUG UGA AGU UCA UTT-3’ and 5’-AUG AAC UUC ACA UCU GUG GTT-3’ and the negative control were 5’-UUC UCC GAA CGU GUC ACG UTT-3’ and 5’-ACG UGA CAC GUU CGG AGA ATT-3’, respectively. All the siRNA sequences were purchased from Shanghai GenePharma Co., Ltd. (Shanghai, China). Cells were transiently transfected with 50 nM siCCND1 or negative control using GP-transfect-Mate in serum and penicillin–streptomycin free condition according to the manufacturer’s instructions. Briefly, GP-transfect-Mate-medium mixture and RNA oligo-medium mixture were prepared and placed at room temperature for 5 min. Then, the GP-transfect-Mate-medium mixture was added dropwise into RNA oligo-medium mixture and placed at room temperature for 15–20 min before the infection. Then, 24 h after the transfection, cells were treated with insulin and OA alone or in combination as described before.

### Animal Experiment

All the animal studies and procedures were approved by the Animal Ethics Committee of China Pharmaceutical University (License No.: SYXK 2018-0019). Five-week-old male athymic nude mice were purchased from Vital River Laboratory Animal Technology. All the mice were housed in a temperature-controlled specific pathogen-free (SPF) environment (24°C ± 2°C) and kept on a 12-h light/dark cycle, having free access to sterilized food and water. The mice were randomly divided into five groups: control (n = 6); oleic acid treatment (OA, n = 6); insulin treatment (INS, n = 6); oleic acid plus insulin treatment (IO, n = 6); oleic acid, insulin, palbociclib [cyclin-dependent kinase (CDK)4/6 inhibitor] and SCH772984 [extracellular signal-regulated kinase (ERK)1/2 inhibitor] treatment (IOPS, n = 6). Oleic acid and palbociclib were administered intragastrically (i.g.) at a dose of 2.0 g/kg/day and 120 mg/kg/day, respectively. Insulin was administered subcutaneously (s.c.) at a dose of 2.5 U/kg/day. SCH772984 was administered intraperitoneally (i.p.) at a dose of 50 mg/kg/day. In addition, individuals in the groups of INS, IO, and IOPS were given 10% glucose to prevent hypoglycemia. Before the injection of HCT116 cells, individuals in the OA and INS groups were pretreated with oleic acid and insulin respectively, and mice in the IO and IOPS groups were pretreated with both oleic acid and insulin for 10 days to build a high oleic acid and insulin environment.

On day 0, HCT116 cells (~1 × 10^7^) suspended in 0.2 ml serum-free DMEM were subcutaneously inoculated in the right flank region. Six days later when colon carcinoma cells were confirmed to plant successfully, we continued to treat controls with vector, OA with oleic acid, INS with insulin, IO with insulin and oleic acid, and IOPS with insulin, oleic acid, palbociclib, and SCH772984. Tumor volume (TV = length × width^2^/2) and body weight were monitored every day. Mice were euthanized when the tumor volume of the IO group reached 2,000 mm^3^. Blood and tumor tissue samples were collected for further experiments.

### Immunohistochemistry and Hematoxylin and Eosin Staining

Tumor tissues were fixed in 4% paraformaldehyde for 3 days and then embedded in paraffin. Hematoxylin and eosin (H&E) staining was performed to observe histopathological features of the tumor. The expression of Ki67, p-ERK1/2, c-Myc, and cyclin D1 were detected by immunohistochemistry (IHC). IHC and H&E staining were performed by professionals in Jiangsu Hospital of Integrated Traditional and Western Medicine (Nanjing, China).

### Statistical Analysis

Statistical analysis was performed using GraphPad Prism 8.0 software (GraphPad Software Inc., San Diego, USA), and the results were expressed as mean ± SD. Each experiment was performed at least three times independently. All the data were statistically analyzed using Student’s t-test unless otherwise noted. p < 0.05 was considered significantly different.

## Results

### Insulin and Oleic Acid Served as the Bridge Between Type 2 Diabetes Mellitus and Colorectal Cancer

**Figure 1:**
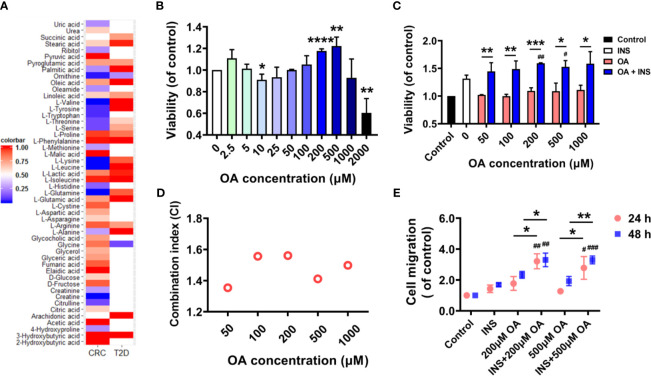
Insulin and oleic acid (OA) synergistically promoted the proliferation and migration of colon carcinoma cell line HCT116. **(A)** Heatmap of common dysregulated metabolites found in both colorectal cancer (CRC) and type 2 diabetes mellitus (T2D) based on reported metabolomics data. **(B)** OA promoted the proliferation of HCT116 in FBS-free condition. ^*^p < 0.05, ^**^p < 0.01, and ^***^p < 0.001 compared with control. **(C)** Cell viability of HCT116 in insulin (50 nM) environment. X axis represents the concentration of OA. ^*^p < 0.05, ^**^p < 0.01, and ^***^p < 0.001 compared with OA. ^#^p < 0.05, ^##^p < 0.01, and ^###^p < 0.001 compared with INS. **(D)** Combination index analysis of insulin (50 nM) and OA (50, 100, 200, 500, and 1,000 μM). CI < 0.85 defines an antagonistic effect, CI > 1.15 a synergistic effect, and CI 0.85–1.15 a nearly additive effect. **(E)** Wound healing assay to investigate the migration ability of HCT116 under insulin and OA alone or in combination treatments. ^*^p < 0.05, ^**^p < 0.01, ^***^p < 0.001, and ****p < 0.0001 compared with OA; ^#^p < 0.05, ^##^p < 0.01, and ^###^p < 0.001 compared with INS.

In total, 22 metabolites were found dysregulated in both T2D and CRC based on published metabolomics studies. A heatmap showing the relative concentration of these metabolites compared to controls was generated (**Figure 1A**). We then explored whether these metabolites could promote the development of CRC by supplementing them to the cell culture medium of HCT116, and an MTT assay was applied to check the cell viability. As a result, only OA slightly promoted the proliferation of HCT116 and did not show obvious cell toxicity even at 1,000 μM. The rest of the metabolites showed almost no effects (**Figure S1A**). The other three fatty acids (LOA, PA, and SA) in the list were confirmed to have no similar effects as OA did (**Figure S1B**). Existing metabolomic papers related to the dysregulation of OA in CRC and T2D are summarized in **Table S1**. We then treated the cells in serum-free condition for 48 h to check whether the existence of FBS affected the proliferation of HCT116. Surprisingly, OA promoted the proliferation of HCT116 much more significantly without FBS at the concentration of 200 or 500 μM (**Figure 1B**).

It is known that hyperglycemia and insulin resistance are the typical features of T2D. We therefore further explored the effect of high glucose and insulin on OA-induced promotion of cell proliferation. Glucose at concentrations of 5.5 and 11 mM was used to simulate physiological and diabetogenic conditions, respectively (16). It was found that the promotion effect of OA was increased remarkably in the presence of 50 nM of insulin (**Figure 1C**). However, high glucose showed no similar effect (**Figure S1C**). In addition, no further promotion was observed under both high insulin (50 nM) and high glucose condition compared to only high insulin (**Figure S1D**). Herein, we assumed that insulin and OA might have a synergistic effect in promoting the proliferation of HCT116. To prove this, we referred to Jin’s formula (21) to calculate the combination index of insulin (50 nM) and OA (50, 100, 200, 500, and 1,000 μM). A synergistic effect is usually acknowledged when the combination index exceeds 1.15. The results showed that the combination indices at different OA concentrations were all above 1.35 (**Figure 1D**). We then further confirmed that insulin and OA promoted the migration of HCT116 synergistically by wound healing assay (**Figures 1E** and **S2**). Taken together, our results showed that the combination of insulin and OA synergistically promoted the proliferation and migration of HCT116. Insulin and OA may serve as the bridge between T2D and CRC.

### P-ERK1/2 and Cyclin D1 Served as the Crosstalk in Insulin and Oleic Acid-Mediated Colorectal Cancer Deterioration Process

**Figure 2:**
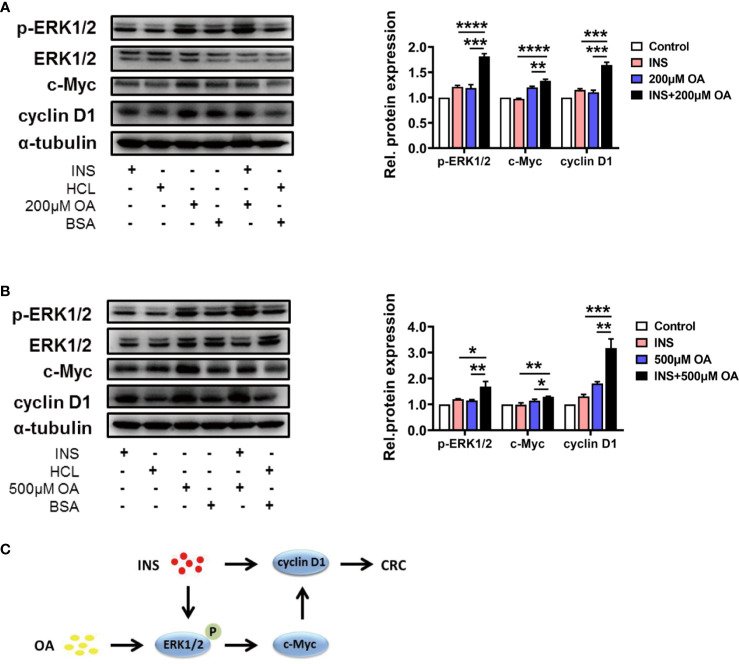
Insulin and oleic acid (OA) promoted the development of colorectal cancer (CRC) synergistically through ERK/c-Myc/cyclin D1 pathway. Protein level of p-ERK1/2, ERK1/2, c-Myc, and cyclin D1 treated with **(A)** insulin (50 nM) and/or OA 200 μM and **(B)** 500 μM were measured. Alpha-tubulin served as an internal control. Groups HCl, BSA, and HCl+BSA are the control of INS, OA, and INS+OA, respectively. **(C)** Pathway diagram of the synergistic effect of insulin and oleic acid in CRC progression. ^*^p < 0.05, ^**^p < 0.01, ^***^p < 0.001, and ****p < 0.0001.

Based on the results obtained above, we investigated the key pathways involved in the process of insulin and OA synergistically promoting the proliferation and migration of HCT116. Kyoto Encyclopedia of Genes and Genomes (KEGG) database indicates MEK1/2/ERK1/2/c-Myc/cyclin D1 is a canonical pathway during the occurrence and development of CRC, and we suspected that this pathway may be involved in the synergistic effect of insulin and OA. As a result, we found that insulin and OA upregulated the expression of cyclin D1 and showed a synergistic effect (**Figures 2A, B** **)**. Based on this, we explored whether the upstream of cyclin D1 was also regulated, and the results showed that insulin can upregulate the expression of p-ERK1/2, but not c-Myc (**Figures 2A, B** **)**. This indicated that insulin did not act directly on c-Myc. Interestingly, we discovered that OA could upregulate the expression of all the three proteins and that the combination of insulin and OA showed more significant effects (**Figures 2A, B** **)**. In summary, we confirmed that OA could promote CRC development *via* the ERK1/2/c-Myc/cyclin D1 pathway (**Figures 2A, B** **)**. Meanwhile, insulin aggravated the effect of OA by acting on p-ERK1/2 and cyclin D1. Two vital proteins, p-ERK1/2 and cyclin D1, as the crosstalk between insulin and OA-mediated pathway, promoted CRC deterioration together (**Figure 2C**).

### P-ERK1/2 Inhibition Blocked the Proliferation and Migration of HCT116 Induced by Insulin and Oleic Acid

**Figure 3:**
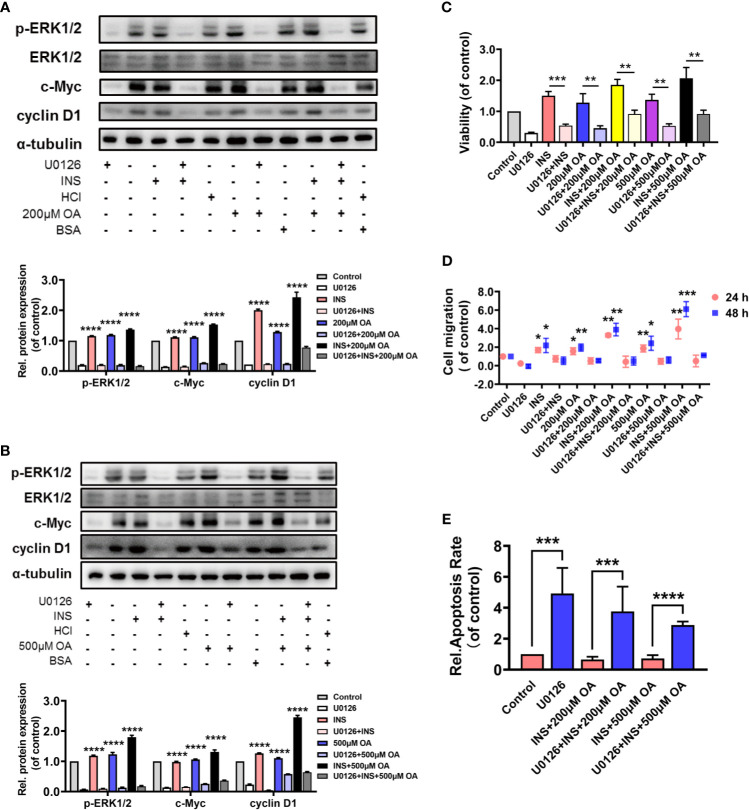
U0126 blocked the carcinogenic effect of insulin and/or oleic acid (OA) *via* ERK1/2/c-Myc/cyclin D1 pathway in HCT116. Protein expression level of **(A, B)** p-ERK1/2, ERK1/2, c-Myc, and cyclin D1. **(C)** Cell viability, **(D)** cell migration, and **(E)** cell apoptosis assay were performed on HCT116 that were pretreated with U0126 (10 μM) for 2 h before insulin (50 nM) and/or OA (200 or 500 μM) stimulation. ^*^p < 0.05, ^**^p < 0.01, ^***^p < 0.001, and ****p < 0.0001 compared with corresponding groups with U0126 treatment.

A MEK1/2 inhibitor, U0126, was applied to verify our findings. HCT116 was pretreated with U0126 for 2 h before insulin treatment, and the results showed that U0126 significantly decreased p-ERK1/2, c-Myc, and cyclin D1 expressions that were highly increased with OA and insulin treatment (**Figures 3A, B** **)**. On the other hand, U0126 decreased the cell viability (**Figure 3C** and **Figure S3**) and cell migration rate (**Figure 3D** and **Figure S4**) and increased the cell apoptosis remarkably (**Figure 3E** and **Figure S5**). These results indicated p-ERK1/2 inhibition partially prevented the deterioration of CRC induced by insulin and OA and promoted cell apoptosis, indicating that p-ERK1/2 might be a potential target for T2D-associated CRC treatment.

### Silence of Cyclin D1 Reverted the Proliferation and Migration of HCT116 Induced by Insulin and Oleic Acid

**Figure 4:**
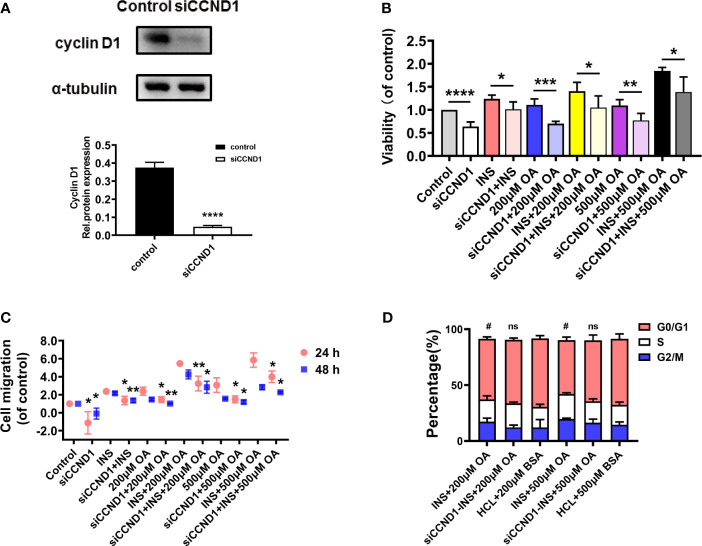
Cyclin D1 knockdown inhibited the oncogenic phenotypes of HCT116. **(A)** Cyclin D1 relative expression level after CCND1 knockdown using siRNA. **(B)** Cell viability, **(C)** cell migration in 24 and 48 h, and **(D)** cell cycle were detected in HCT116 after cyclin D1 knockdown using siRNA before insulin (50 nM) and/or OA (200 or 500 μM) stimulation. ^*^p < 0.05, ^**^p < 0.01, ^***^p < 0.001, and ****p < 0.0001 compared with the corresponding groups without siCCND1. ^#^p < 0.05 compared with corresponding controls; ns, not significant.

To further verify our findings, cyclin D1 was silenced to investigate the proliferation and migration index of HCT116. The results showed that the protein expression level of cyclin D1 was significantly downregulated after the transfection, proving that the target sequence was effective (**Figure 4A**). The MTT results showed that cell viability decreased significantly when cyclin D1 was silenced (**Figure 4B**), and the wound healing assay showed that the cell migration rate also decreased (**Figure 4C** and **Figure S6**). In addition, we investigated the potential changes in the cell cycle as cyclin D1 is a cell cycle protein and mediates the cell cycle G1–S transition. As a result, insulin and OA combination significantly shortened cell G0/G1 phase compared with control (p < 0.05). However, cyclin D1 silence induced G0/G1 phase arrest, and there was no significant difference between the siRNA intervention group and the controls (**Figure 4D** and **Figure S7**). These results proved that insulin and OA upregulated the expression of cyclin D1 and promoted cell G1–S phase transition to accelerate the tumor growth, and the silence of cyclin D1 reverted this effect.

### ERK Inhibitor SCH772984 and CDK4/6 Inhibitor Palbociclib Prevented the HCT116 Proliferation Induced by Insulin and Oleic Acid *In Vitro*

**Figure 5:**
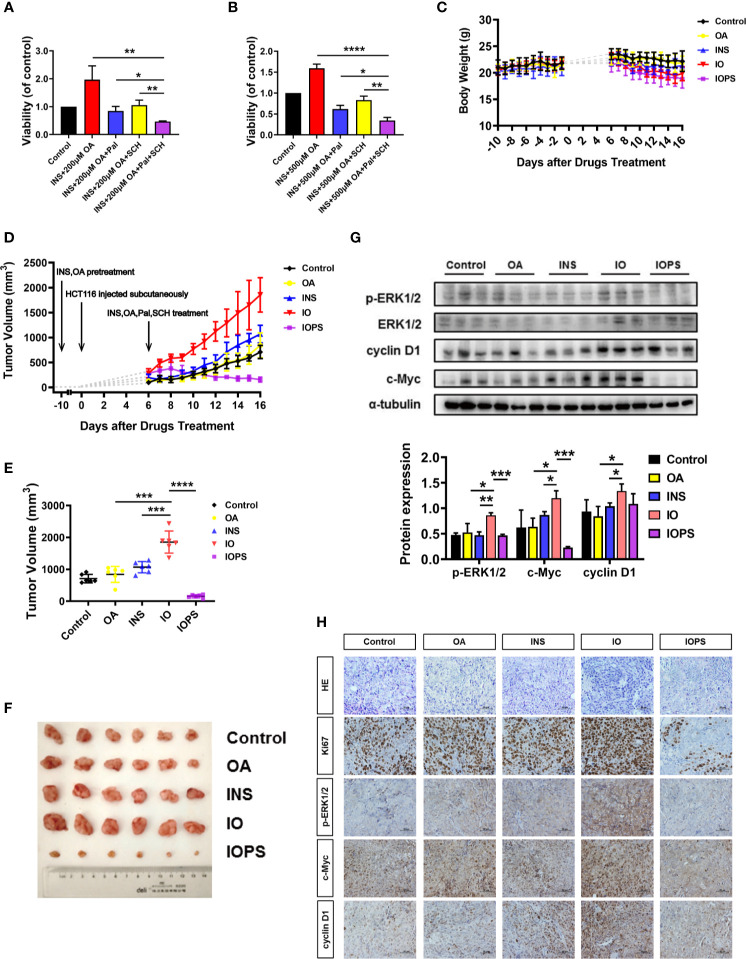
ERK1/2 and CDK4/6 inhibitors performed significant antitumor effect *in vitro* and insulin and oleic acid (OA) aggravated tumor growth *via* ERK1/2/c-Myc/cyclin D1 in a colorectal cancer (CRC) xenograft model. Cell viability when HCT116 cells were treated with palbociclib (4 or 6 μM) and SCH772984 (70 or 130 nM) in **(A)** insulin (50 nM) and OA 200 μM or **(B)** 500 μM condition; each experiment was performed three times independently. **(C)** Body weight and **(D)** tumor volume monitor of each group across the whole experiment. **(E)** Tumor volume at the end of the experiment. **(F)** Photos of subcutaneous tumor at the end of the experiment. **(G)** Western blot analysis of p-ERK1/2, c-Myc, and cyclin D1 in tumors. **(H)** H&E staining and immunohistochemistry (IHC) analysis of Ki67, p-ERK1/2, c-Myc, and cyclin D1 in tumors. The scale was 400×. ^*^p < 0.05, ^**^p < 0.01, ^***^p < 0.001, and ****p < 0.0001.

Although U0126 significantly hindered the proliferation and migration of HCT116, there is the issue of drug resistance, and it has been reported that ERK1/2 inhibitor is effective in the treatment of MEK1/2 inhibitor resistance (22). Therefore, ERK1/2 inhibitor SCH772984 was chosen for the *in vivo* validation. For another vital protein cyclin D1 in the pathway, as it does not show enzymatic activity until formed with CDK4 or CDK6 into active complexes (23), and silencing cyclin D1 *in vivo* is challenging, an approved CDK4/6 inhibitor (palbociclib) was selected to inhibit cyclin D1/CDKs axis. Before the animal experiment, we validated the antitumor effect of SCH772984 and palbociclib *in vitro*. The IC_50_ of palbociclib and SCH772984 in normal conditions (no insulin and OA) was 1.6 μM and 23.9 nM, respectively, while the values were much higher when the cells were treated with insulin and OA (3.8 μM and 72.1 nM with 50 nM of insulin and 200 μM of OA, 5.9 μM and 132.9 nM with 50 nM of insulin and 500 μM of OA; **Figure S8**). In addition, palbociclib and SCH772984 showed a cell suppression effect under high insulin and OA condition (**Figures 5A, B** **)**. These results proved that insulin and OA could promote CRC cell proliferation and induce drug resistance. Importantly, ERK1/2 inhibitor SCH772984 and CDK4/6 inhibitor palbociclib were proven to have a significant antitumor effect *in vitro*.

### Insulin and Oleic Acid-Simulated Type 2 Diabetes Mellitus Environment Promoted the Development of Colorectal Cancer in Xenograft Mice

To investigate the effect of high insulin and OA environment on tumor growth in T2D, we established the HCT116 cell xenograft model (**Figure S9**). Body weight (**Figure 5C**) and tumor volume (**Figure 5D**) of each individual were monitored. At the end of the experiment, the tumor volume of the individuals in the OA group showed almost no difference compared with the controls. The tumor volume of the INS individuals was bigger than that of the controls, and in the IO group, it was much bigger than that of any other group (**Figure 5E**). These results indicated that OA alone almost had no effect on cancer cell proliferation *in vivo*, and insulin alone promoted the proliferation of tumor cells to some extent. Importantly, the combination of insulin and OA (IO group) had a more severe influence on solid tumor formation as well as its growth. In addition, to validate the mechanism we speculated from *in vitro* experiments *in vivo*, CDK4/6 and ERK1/2 inhibitors (palbociclib and SCH772984, respectively) were administered (IOPS group). The results showed that the combination of the two inhibitors significantly suppressed the stimulated proliferation of HCT116 xenografts by insulin and OA (**Figures 5E, F** **)**, and the tumor shrank remarkably and tended to become smaller as time went (**Figure 5D**). This suggests that the cancer cell activity was reduced, and the p-ERK1/2 and cyclin D1/CDK4/6 axis was involved in the development of CRC.

Furthermore, IHC and Western blot showed that the level of p-ERK1/2, c-Myc, and cyclin D1 increased significantly in IO individuals, and the results were in accordance with the above *in vitro* experiments (**Figures 5G, H** **)**. Also, Ki67, a marker of cell proliferation and tumor deterioration, was highly expressed in the IO group. And for IOPS individuals, Ki67 was downregulated remarkably (**Figure 5H**). Taken together, insulin and OA promoted colon carcinoma cell colonization and outgrowth in the progress of CRC occurrence and development and performed a synergistic oncogenic effect *in vivo*. The combination of palbociclib and SCH772984 might be a promising means for clinical T2D-associated CRC therapy.

## Discussion

Epidemiology studies showed that diabetes could cause many health problems, such as cardiovascular disease (24), Alzheimer’s disease (25), and multiple types of cancers. While it has been reported that T2D is strongly associated with CRC, the underlying molecular mechanisms were poorly understood. In this study, by utilizing a functional metabolomics strategy, we found that insulin and OA promoted the development of CRC synergistically *via* the ERK1/2/c-Myc/cyclin D1 pathway.

OA (C18:1), an endogenous monounsaturated fatty acid, is one of the upregulated metabolites in the T2D population (26, 27). It has been shown that abnormally elevated OA level was associated with the development of cervical cancer (28), breast cancer (29), prostate cancer (30), and liver cancer (31), as cancer cells require fatty acids for membrane proliferation, energy storage, and signaling molecule generation (28). Studies showed that OA induced the activation of Src kinase and its downstream ERK1/2 *via* a CD36-dependent manner to promote cervical cancer cell growth and metastasis (28). Another pathway, phosphatase and tensin homolog (PTEN)/mTOR/nuclear factor kappa B (NF-κB), is also mediated by OA in the process of hepatoma proliferation and progression (31). In our study, OA was proven to promote the progression of CRC. More importantly, the oncogenic effect was more significant under high insulin conditions.

Insulin resistance, defined as a defect in the capacity of insulin to drive glucose to its target tissue, is one of the major characteristics of T2D (32). In other words, it is the condition in which the cell, tissue, or organism fails to respond to insulin effectively (33). As a result, insulin compensatory secretion is initiated. It is reported that insulin has profound metabolic and mitogenic effects through binding to different receptors (34). Multiple studies revealed that insulin promoted tumor growth by upregulating the expression of p-AKT and p-ERK1/2 (35), which are crucial proteins during the process of cancer deterioration. It has also been reported that hyperinsulinemia could activate the insulin receptor (IR)/insulin-like growth factor 1 receptor (IGF-1R) signaling pathway to promote the development of breast cancer (36). In the current study, we discovered that insulin promoted CRC deterioration *in vitro* and *in vivo*, particularly, with the presence of OA, insulin showed a more powerful tumor-promoting effect. As metabolic reprogramming has been suggested as a key hallmark of cancer progression (37), we speculated that the pathological characteristics of T2D provided a favorable environment for CRC deterioration. As T2D has many other pathological features (chronic low-grade inflammation, glucose and lipid metabolism dysregulation, etc.), there are possibly other pathways mediating T2D-associated CRC.

Mitogen-activated protein kinase (MAPK) cascades play an important role in the regulation of cell proliferation, survival, and differentiation under physiological conditions (38). However, MAPK cascades were found activated aberrantly in multiple cancers (39). Therefore, inhibitors acting on these cascades have caught extensive attention as they hold great potential in cancer therapeutics (38). The KEGG database indicates that the MEK1/2/ERK1/2/c-Myc/cyclin D1 is an important pathway in the development of CRC. In this pathway, ERK1/2 is activated by the phosphorylation of the Thr202/Tyr204 site and subsequently phosphorylates numerous proteins involved in multiple malignant phenotypic features and ultimately aggravates cancer deterioration (40).

Another crucial protein, cyclin D1, a downstream protein of c-Myc to drive cell G1-to-S phase transition, plays an important role in normal cell proliferation and aberrant tumor cell diffusion (23). It has been reported that abnormally expressed cyclin D1 was associated with multiple cancers, such as pancreatic cancer (41), non-small-cell lung carcinoma (42), breast cancer (43), head and neck squamous cell carcinoma (44), endometrial cancer (45), and colorectal carcinoma (46). Studies on cyclin D1 showed that this protein does not show enzymatic activity until it forms active complexes with CDK4 or CDK6 (23). Therefore, inhibiting CDK4/6 is a potential means to inhibit tumor growth. Selective CDK4/6 inhibitors such as palbociclib, ribociclib, and abemaciclib have achieved curative effects in breast cancer (47). As a first-in-class CDK4/6 inhibitor, palbociclib has been introduced for the treatment of estrogen receptor-positive, human epidermal growth factor receptor 2-negative metastatic breast cancer (48), but it has the side effects of myelosuppression and fatigue (49). Side effects were also found in our study. Adverse effects including weakness, weight loss, diarrhea, hematochezia, and hematuria were observed in the IOPS mice. However, the side effects were very mild, and not all of them occurred in each individual. We speculated that the gastrointestinal tract response might correlate with the high doses applied. Although we discovered that the combination of ERK1/2 and CDK4/6 inhibitors had a profound effect on CRC treatment *in vitro* and *in vivo*, the effect of palbociclib or SCH772984 alone *in vivo* is yet to be investigated.

It has been reported that there are six key driver genes, namely, APC, KRAS, BRAF, PIK3CA, SMAD4, and p53, in CRC. The cell line HCT116 used in our study primarily has mutations in KRAS and PIK3CA. Similar experiments should be performed on other CRC cell lines that harbor distinct mutations to investigate whether the findings are consistent. In conclusion, existing human metabolomics data were utilized to find common dysregulated metabolites in CRC and T2D, and OA was confirmed to promote the development of colon carcinoma cell HCT116. Further study revealed that insulin and OA synergistically promoted the deterioration of CRC *in vitro* and *in vivo*, and this effect was accomplished through acting on the ERK1/2/c-Myc/cyclin D1 pathway. In addition, we proved that ERK1/2 and CDK4/6 might be promising therapeutic targets against T2D-associated CRC in the clinic.

## Data Availability Statement

The original contributions presented in the study are included in the article/**Supplementary Material**. Further inquiries can be directed to the corresponding authors.

## Ethics Statement

The animal study was reviewed and approved by Animals Ethics Committee of China Pharmaceutical University.

## Author Contributions

YZ and FX conceived the project. PZ and FX supervised the project. YZ, DW, BL, XH, QL, CL, and RX conducted the experiments. YZ and PZ analyzed the data and drafted the manuscript. FX, PZ, and YXZ edited and reviewed the manuscript. All authors contributed to the article and approved the submitted version.

## Funding

This study was supported by the NSFC (No. 82073812, 81773682, 81773861), Jiangsu Provincial National Science Foundation for Distinguished Young Scholars (No. BK20180027), National Science and Technology Major Project (2017ZX09101001), the Fundamental Research Funds for the Central Universities (2632021PY03), Double First-Class University project, the Program for Jiangsu province Innovative Research Team, and a project funded by the Priority Academic Program Development of Jiangsu Higher Education Institutions (PAPD).

## Conflict of Interest

The authors declare that the research was conducted in the absence of any commercial or financial relationships that could be construed as a potential conflict of interest.

## Publisher’s Note

All claims expressed in this article are solely those of the authors and do not necessarily represent those of their affiliated organizations, or those of the publisher, the editors and the reviewers. Any product that may be evaluated in this article, or claim that may be made by its manufacturer, is not guaranteed or endorsed by the publisher.

## References

[1] Available at: https://gco.iarc.fr/today/home.

[2] Kvietkauskas M Zitkute V Leber B Strupas K Stiegler P Schemmer P. The Role of Melatonin in Colorectal Cancer Treatment: A Comprehensive Review. Ther Adv Med Oncol (2020) 12:1–14. 10.1177/1758835920931714 PMC7370547 32733605

[3] Stoffel EM Murphy CC. Epidemiology and Mechanisms of the Increasing Incidence of Colon and Rectal Cancers in Young Adults. Gastroenterology (2020) 158:341–53. 10.1053/j.gastro.2019.07.055 PMC6957715 31394082

[4] Fagunwa IO Loughrey MB Coleman HG. Alcohol, Smoking and the Risk of Premalignant and Malignant Colorectal Neoplasms. Best Pract Res Cl Ga (2017) 31:561–8. 10.1016/j.bpg.2017.09.012 29195676

[5] Bardou M Barkun AN Martel M. Obesity and Colorectal Cancer. Gut (2013) 62:933–47. 10.1136/gutjnl-2013-304701 23481261

[6] Nadeem MS Kumar V Al-Abbasi FA Kamal MA Anwar F. Risk of Colorectal Cancer in Inflammatory Bowel Diseases. Semin Cancer Biol (2020) 64:51–60. 10.1016/j.semcancer.2019.05.001 31112753

[7] Luo S Li J-Y Zhao L-N Yu T Zhong W Xia Z-S. Diabetes Mellitus Increases the Risk of Colorectal Neoplasia: An Updated Meta-Analysis. Clin Res Hepatol Gastroenterol (2016) 40:110–23. 10.1016/j.clinre.2015.05.021 26162991

[8] Eibl G Cruz-Monserrate Z Korc M Petrov MS Goodarzi MO Fisher WE. Diabetes Mellitus and Obesity as Risk Factors for Pancreatic Cancer. J Acad Nutr Diet (2018) 118:555–67. 10.1016/j.jand.2017.07.005 PMC5845842 28919082

[9] Printz C. Diabetes Associated With Increased Risk of Liver Cancer. Cancer (2014) 120:1288. 10.1002/cncr.28718 24756959

[10] Del Puerto-Nevado L Minguez P Corton M Solanes-Casado S Prieto I Mas S. Molecular Evidence of Field Cancerization Initiated by Diabetes in Colon Cancer Patients. Mol Oncol (2019) 13:857–72. 10.1002/1878-0261.12438 PMC6441931 30628165

[11] Maskarinec G Fontaine A Torfadottir JE Lipscombe LL Lega IC Figueroa J. The Relation of Type 2 Diabetes and Breast Cancer Incidence in Asian, Hispanic and African American Populations—A Review. Can J Diabetes (2018) 42:100–5. 10.1016/j.jcjd.2017.02.005 28506814

[12] Yang J Nishihara R Zhang X Ogino S Qian ZR. Energy Sensing Pathways: Bridging Type 2 Diabetes and Colorectal Cancer? J Diabetes Complications (2017) 31:1228–36. 10.1016/j.jdiacomp.2017.04.012 PMC5501176 28465145

[13] Petrelli F Ghidini M Rausa E Ghidini A Cabiddu M Borgonovo K. Survival of Colorectal Cancer Patients With Diabetes Mellitus: A Meta-Analysis. Can J Diabetes (2020) 45:186–97. 10.1016/j.jcjd.2020.06.009 33039329

[14] Perry RJ Shulman GI. Mechanistic Links Between Obesity, Insulin, and Cancer. Trends Cancer (2020) 6:75–8. 10.1016/j.trecan.2019.12.003 PMC7214048 32061306

[15] Brzacki V Nagorni A Kallistratos M Manolis A Lovic D. Diabetes Mellitus: A Clinical Condition Associated With Metabolic Syndrome and Colorectal Cancer Risk. Curr Pharmacol Rep (2019) 5:205–9. 10.1007/s40495-019-00183-8

[16] Tomas N Masur K Piecha J Niggemann B Zänker K. Akt and Phospholipase Cγ are Involved in the Regulation of Growth and Migration of MDA-MB-468 Breast Cancer and SW480 Colon Cancer Cells When Cultured With Diabetogenic Levels of Glucose and Insulin. BMC Res Notes (2012) 5:214. 10.1186/1756-0500-5-214 22554284 PMC3393613

[17] Uchiyama K Yagi N Mizushima K Higashimura Y Hirai Y Okayama T. Serum Metabolomics Analysis for Early Detection of Colorectal Cancer. J Gastroenterol (2016) 52:677–94. 10.1007/s00535-016-1261-6 27650200

[18] Budhathoki S Iwasaki M Yamaji T Yamamoto H Kato Y Tsugane S. Association of Plasma Concentrations of Branched-Chain Amino Acids With Risk of Colorectal Adenoma in a Large Japanese Population. Ann Oncol (2017) 28:818–23. 10.1093/annonc/mdw680 28011449

[19] Rebholz CM Yu B Zheng Z Chang P Tin A Köttgen A. Serum Metabolomic Profile of Incident Diabetes. Diabetologia (2018) 61:1046–54. 10.1007/s00125-018-4573-7 PMC5878141 29556673

[20] Cousin SP Hugl SR Wrede CE Kajio H Myers MG Rhodes CJ. Free Fatty Acid-Induced Inhibition of Glucose and Insulin-Like Growth Factor I-Induced Deoxyribonucleic Acid Synthesis in the Pancreatic Beta-Cell Line INS-1. Endocrinology (2001) 142:229–40. 10.1210/endo.142.1.7863 11145586

[21] Han L Dai S Li Z Zhang C Wei S Zhao R. Combination of the Natural Compound Periplocin and TRAIL Induce Esophageal Squamous Cell Carcinoma Apoptosis *In Vitro* and *In Vivo*: Implication in Anticancer Therapy. J Exp Clin Cancer Res (2019) 38:1–17. 10.1186/s13046-019-1498-z 31864387 PMC6925860

[22] Morris EJ Jha S Restaino CR Dayananth P Zhu H Cooper A. Discovery of a Novel ERK Inhibitor With Activity in Models of Acquired Resistance to BRAF and MEK Inhibitors. Cancer Discovery (2013) 3:742–50. 10.1158/2159-8290.CD-13-0070 23614898

[23] Qie S Diehl JA D1 C. Cancer Progression, and Opportunities in Cancer Treatment. J Mol Med (2016) 94:1313–26. 10.1007/s00109-016-1475-3 PMC5145738 27695879

[24] Glovaci D Fan W Wong ND. Epidemiology of Diabetes Mellitus and Cardiovascular Disease. Curr Cardiol Rep (2019) 21:1–8. 10.1007/s11886-019-1107-y 30828746

[25] Jash K Gondaliya P Kirave P Kulkarni B Sunkaria A Kalia K. Cognitive Dysfunction: A Growing Link Between Diabetes and Alzheimer’s Disease. Drug Dev Res (2020) 81:144–64. 10.1002/ddr.21579 31820484

[26] Abu Bakar MH Sarmidi MR. Association of Cultured Myotubes and Fasting Plasma Metabolite Profiles With Mitochondrial Dysfunction in Type 2 Diabetes Subjects. Mol Biosyst (2017) 13:1838–53. 10.1039/C7MB00333A 28726959

[27] Zeng M Liang Y Li H Wang B Chen X. A Metabolic Profiling Strategy for Biomarker Screening by GC-MS Combined With Multivariate Resolution Method and Monte Carlo PLS-Da. Anal Methods (2011) 3:438–45. 10.1039/C0AY00518E 32938047

[28] Yang P Su C Luo X Zeng H Zhao L Wei L. Dietary Oleic Acid-Induced CD36 Promotes Cervical Cancer Cell Growth and Metastasis *Via* Up-Regulation Src/ERK Pathway. Cancer Lett (2018) 438:76–85. 10.1016/j.canlet.2018.09.006 30213558

[29] Hardy S St-Onge GG Joly É Langelier Y Prentki M. Oleate Promotes the Proliferation of Breast Cancer Cells *Via* the G Protein-Coupled Receptor Gpr40. J Biol Chem (2005) 280:13285–91. 10.1074/jbc.M410922200 15695516

[30] Liotti A Cosimato V Mirra P Calì G Conza D Secondo A. Oleic Acid Promotes Prostate Cancer Malignant Phenotype *via* the G Protein-Coupled Receptor FFA1/GPR40. J Cell Physiol (2018) 233:7367–78. 10.1002/jcp.26572 PMC13482116 29663374

[31] Vinciguerra M Carrozzino F Peyrou M Carlone S Montesano R Benelli R. Unsaturated Fatty Acids Promote Hepatoma Proliferation and Progression Through Downregulation of the Tumor Suppressor PTEN. J Hepatol (2009) 50:1132–41. 10.1016/j.jhep.2009.01.027 19398230

[32] Palomer X Pizarro-Delgado J Barroso E Vázquez-Carrera M. Palmitic and Oleic Acid: The Yin and Yang of Fatty Acids in Type 2 Diabetes Mellitus. Trends Endocrinol Metab (2018) 29:178–90. 10.1016/j.tem.2017.11.009 29290500

[33] Kang S Tsai LTY Rosen ED. Nuclear Mechanisms of Insulin Resistance. Trends Cell Biol (2016) 26:341–51. 10.1016/j.tcb.2016.01.002 PMC4844850 26822036

[34] Othman EM Altabaa T Hintzsche H Stopper H. IR and IGF-1R Expression Affects Insulin Induced Proliferation and DNA Damage. Toxicol In Vitro (2017) 39:68–74. 10.1016/j.tiv.2016.11.011 27884723

[35] Guo Y Zhu S-l Wu Y-k He Z Chen Y-Q. Omega-3 Free Fatty Acids Attenuate Insulin-Promoted Breast Cancer Cell Proliferation. Nutr Res (2017) 42:43–50. 10.1016/j.nutres.2017.04.008 28633870

[36] Gallagher EJ Fei K Feldman SM Port E Friedman NB Boolbol SK. Insulin Resistance Contributes to Racial Disparities in Breast Cancer Prognosis in US Women. Breast Cancer Res (2020) 22:1–10. 10.1186/s13058-020-01281-y PMC7216707 32393319

[37] Faubert B Solmonson A DeBerardinis RJ. Metabolic Reprogramming and Cancer Progression. Science (2020) 368:1–10. 10.1126/science.aaw5473 PMC7227780 32273439

[38] Roberts PJ Der CJ. Targeting the Raf-MEK-ERK Mitogen-Activated Protein Kinase Cascade for the Treatment of Cancer. Oncogene (2007) 26:3291–310. 10.1038/sj.onc.1210422 17496923

[39] Drosten M Barbacid M. Targeting the MAPK Pathway in KRAS-Driven Tumors. Cancer Cell (2020) 37:543–50. 10.1016/j.ccell.2020.03.013 32289276

[40] Shen Z Zhang C Qu L Lu C Xiao M Ni R. MKP-4 Suppresses Hepatocarcinogenesis by Targeting ERK1/2 Pathway. Cancer Cell Int (2019) 19:1–15. 10.1186/s12935-019-0776-3 30923463 PMC6423746

[41] Garcea G Neal CP Pattenden CJ Steward WP Berry DP. Molecular Prognostic Markers in Pancreatic Cancer: A Systematic Review. Eur J Cancer (2005) 41:2213–36. 10.1016/j.ejca.2005.04.044 16146690

[42] Gautschi O Ratschiller D Gugger M Betticher DC Heighway J. Cyclin D1 in non-Small Cell Lung Cancer: A Key Driver of Malignant Transformation. Lung Cancer (2007) 55:1–14. 10.1016/j.lungcan.2006.09.024 17070615

[43] Roy PG Thompson AM. Cyclin D1 and Breast Cancer. Breast (2006) 15:718–27. 10.1016/j.breast.2006.02.005 16675218

[44] Rothenberg SM Ellisen LW. The Molecular Pathogenesis of Head and Neck Squamous Cell Carcinoma. J Clin Invest (2012) 122:1951–7. 10.1172/JCI59889 PMC3589176 22833868

[45] Moreno-Bueno G Rodríguez-Perales S Sánchez-Estévez C Marcos R Hardisson D Cigudosa JC. Molecular Alterations Associated With Cyclin D1 Overexpression in Endometrial Cancer. Int J Cancer (2004) 110:194–200. 10.1002/ijc.20130 15069681

[46] McKay JA Douglas JJ Ross VG Curran S Murray GI Cassidy J. Cyclin D1 Protein Expression and Gene Polymorphism in Colorectal Cancer. Int J Cancer (2000) 88:77–81. 10.1002/1097-0215(20001001)88:1<77::AID-IJC12>3.0.CO;2-O 10964085

[47] Du Q Guo X Wang M Li Y Sun X Li Q. The Application and Prospect of CDK4/6 Inhibitors in Malignant Solid Tumors. J Hematol Oncol (2020) 13:1–12. 10.1186/s13045-020-00880-8 32357912 PMC7195725

[48] Ettl J Harbeck N. The Safety and Efficacy of Palbociclib in the Treatment of Metastatic Breast Cancer. Expert Rev Anticancer Ther (2017) 17:661–8. 10.1080/14737140.2017.1347506 28649895

[49] Gong J Cho M Yu KW Waisman J Yuan Y Mortimer J. A Single Institution Experience With Palbociclib Toxicity Requiring Dose Modifications. Breast Cancer Res Treat (2017) 168:381–7. 10.1007/s10549-017-4606-9 PMC5838140 29218462

