# Supplementary material for: Oleic Acid and Insulin as Key Characteristics of T2D Promote Colorectal Cancer Deterioration in Xenograft Mice Revealed by Functional Metabolomics
Source: Front Oncol. 2021 Aug 9;11:685059. doi: 10.3389/fonc.2021.685059 (PMC8381473; doi:10.3389/fonc.2021.685059)
Supplement: Supplementary file 1 [file DataSheet_1.docx]

Supplementary Material

## Supplementary Figures


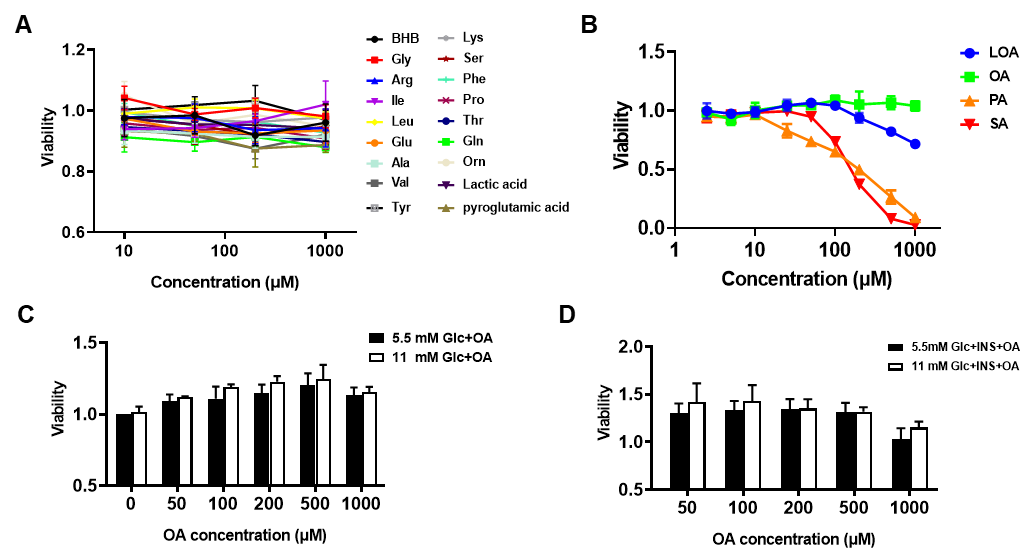


**Figure S1.** Effect of (A) 18 amino acids and (B) 4 fatty acids on the viability of HCT116 cells was evaluated by MTT assay; (C) Effect of high glucose (11 mM) on the OA induced promotion of cell proliferation was evaluated by MTT assay; and (D) Effect of high glucose (11 mM) on the OA induced promotion of cell proliferation at the presence of high insulin (50 nM) was evaluated by MTT assay.


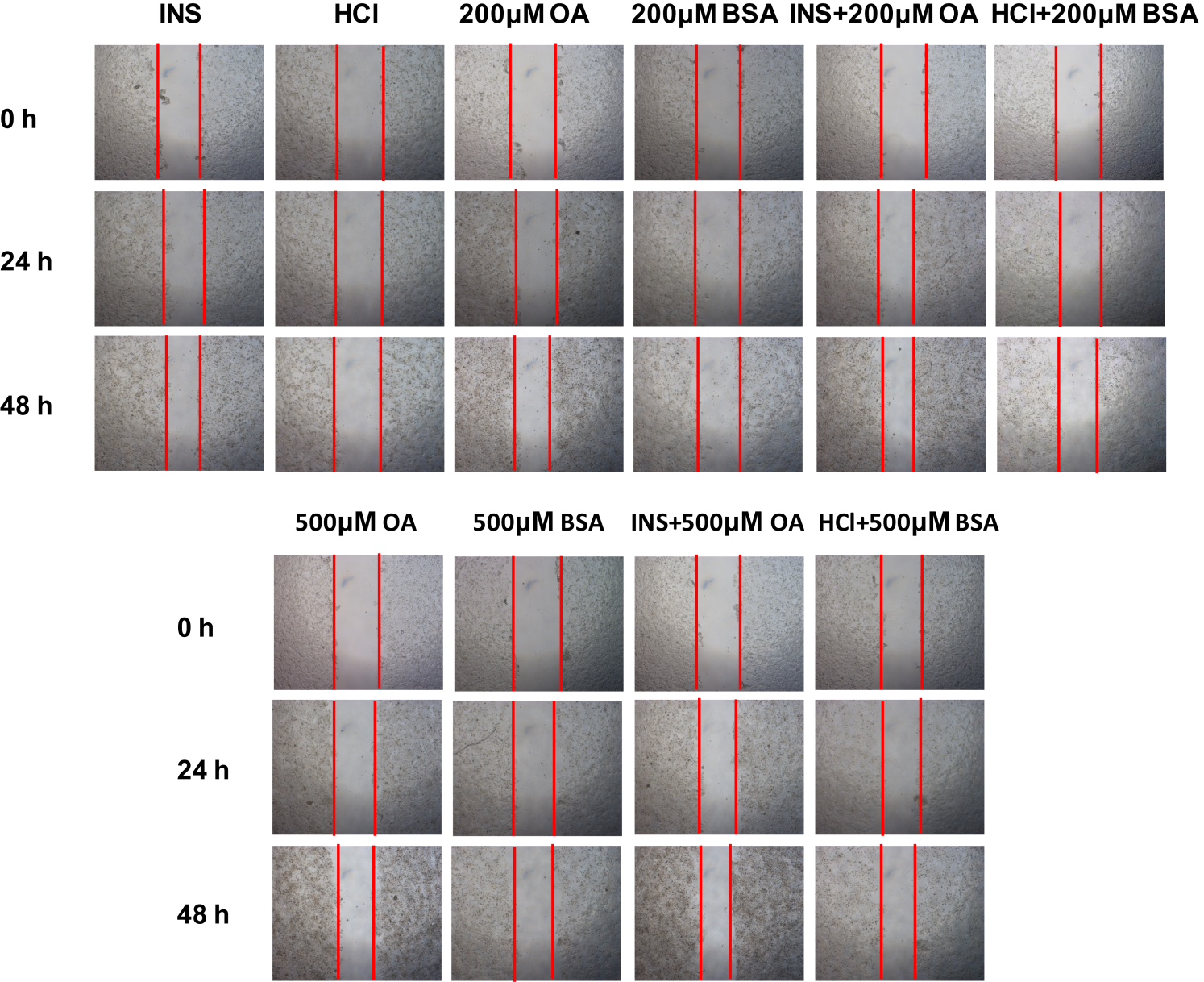


**Figure S2.** Microscopy image of the wound healing assay on HCT116 cells treated with insulin (50 nM) and/or OA (200 or 500 μM).


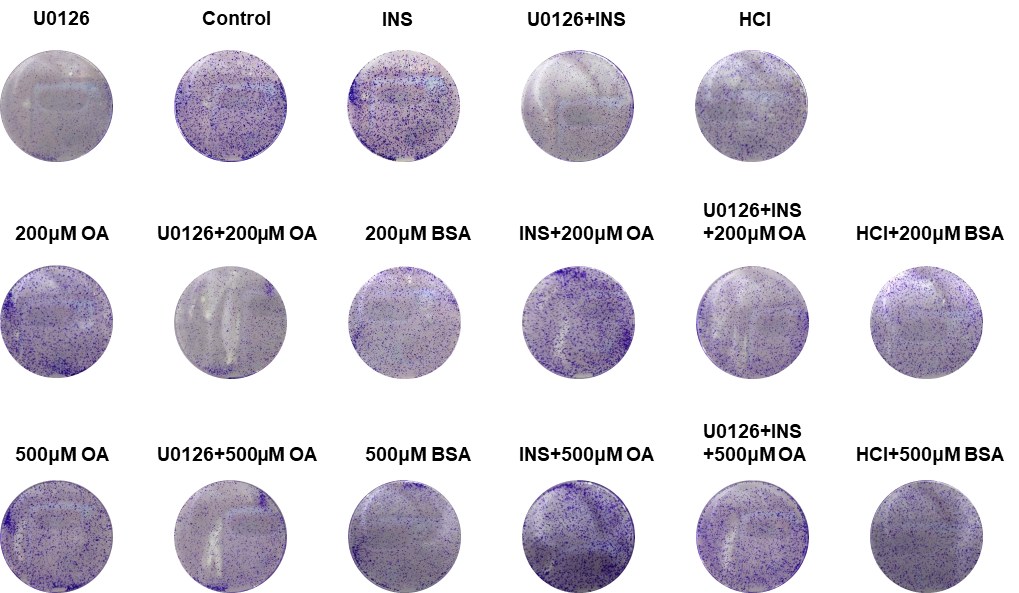


**Figure S3.**  Colony formation assay was performed on HCT116 cells pretreated with U0126 (10 μM) for 2 h before insulin (50 nM) and/or OA (200 or 500 μM) stimulation.


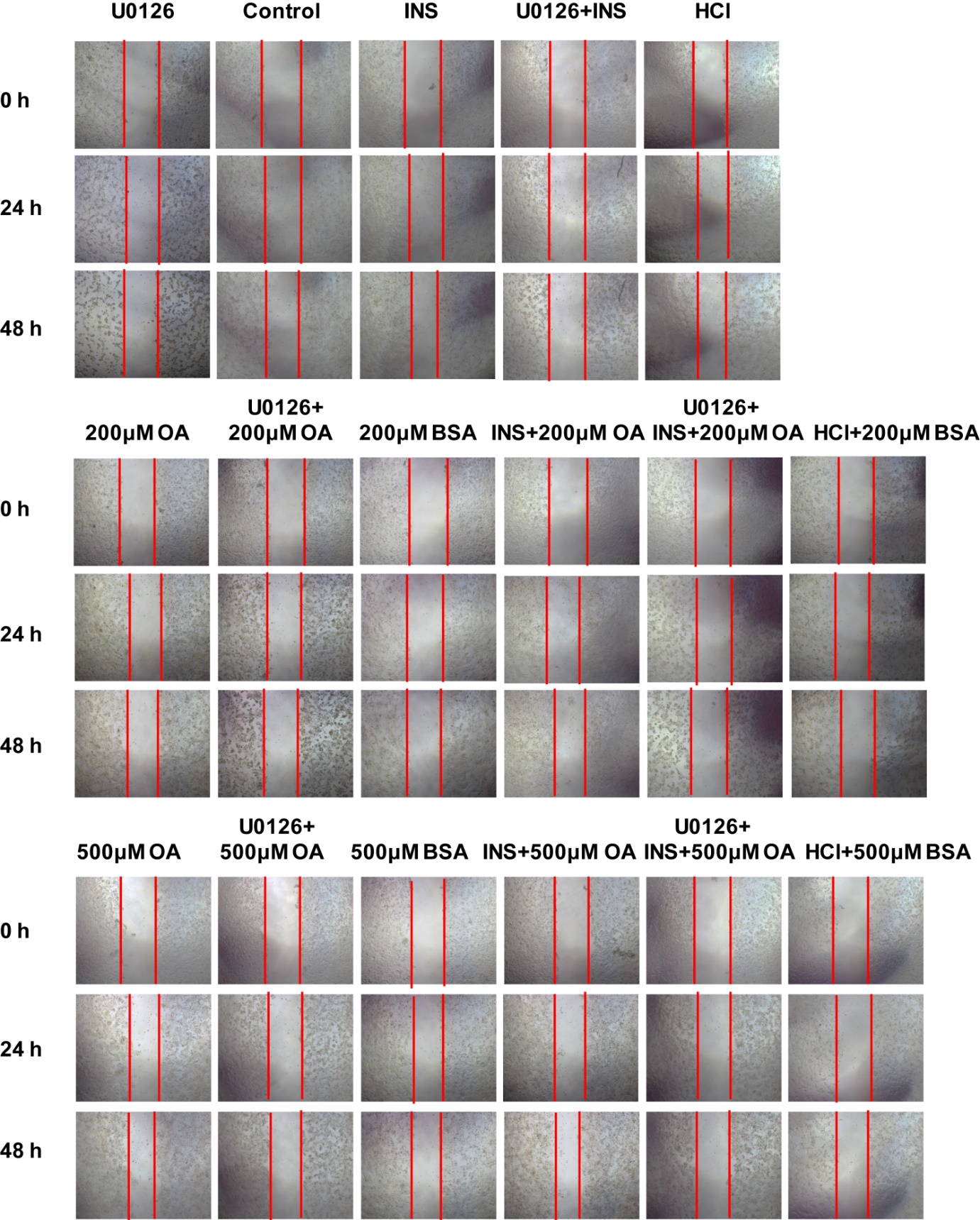


**Figure S4.**  Microscopy image of the wound healing assay on HCT116 cells pretreated with U0126 (10 μM) for 2 h before insulin (50 nM) and/or OA (200 or 500 μM) stimulation.


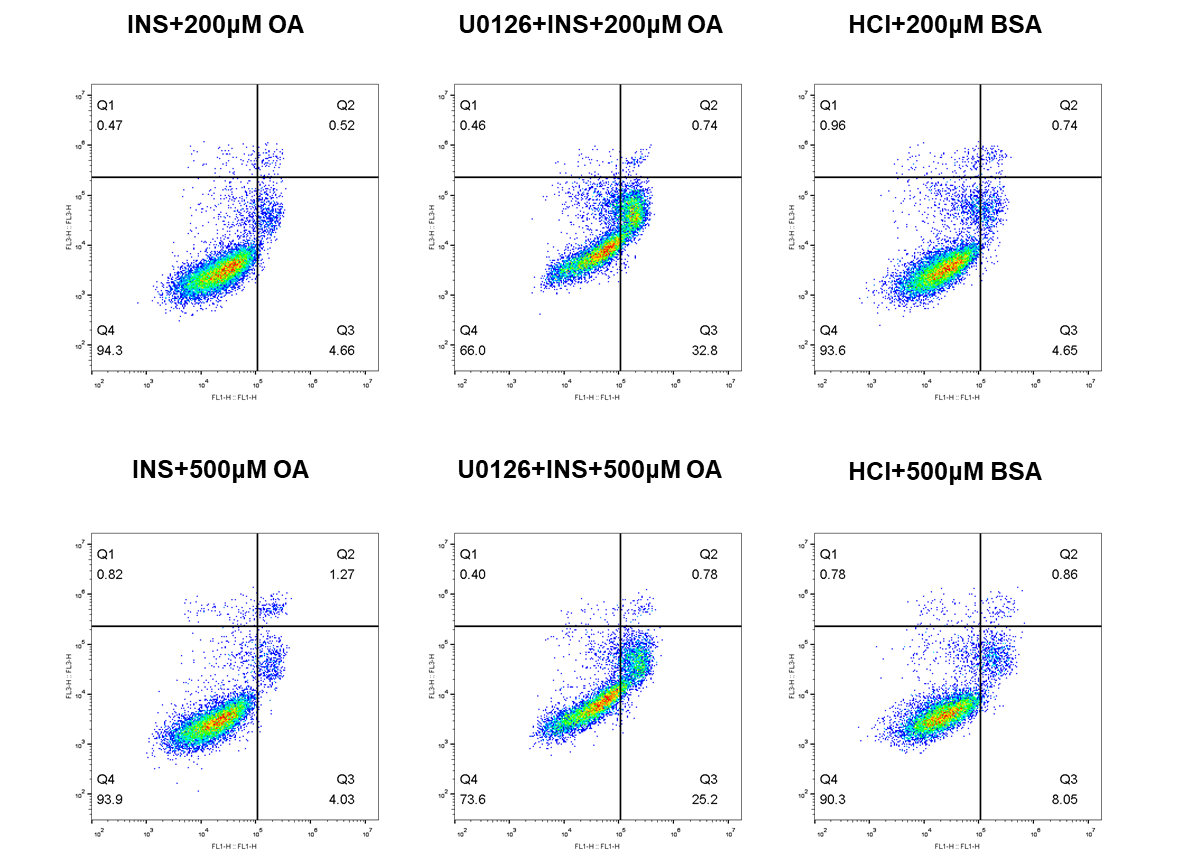


**Figure S5.**  Cell apoptosis assay was performed on HCT116 cells pretreated with U0126 (10 μM) for 2 h before insulin (50 nM) and/or OA (200 or 500 μM) stimulation.


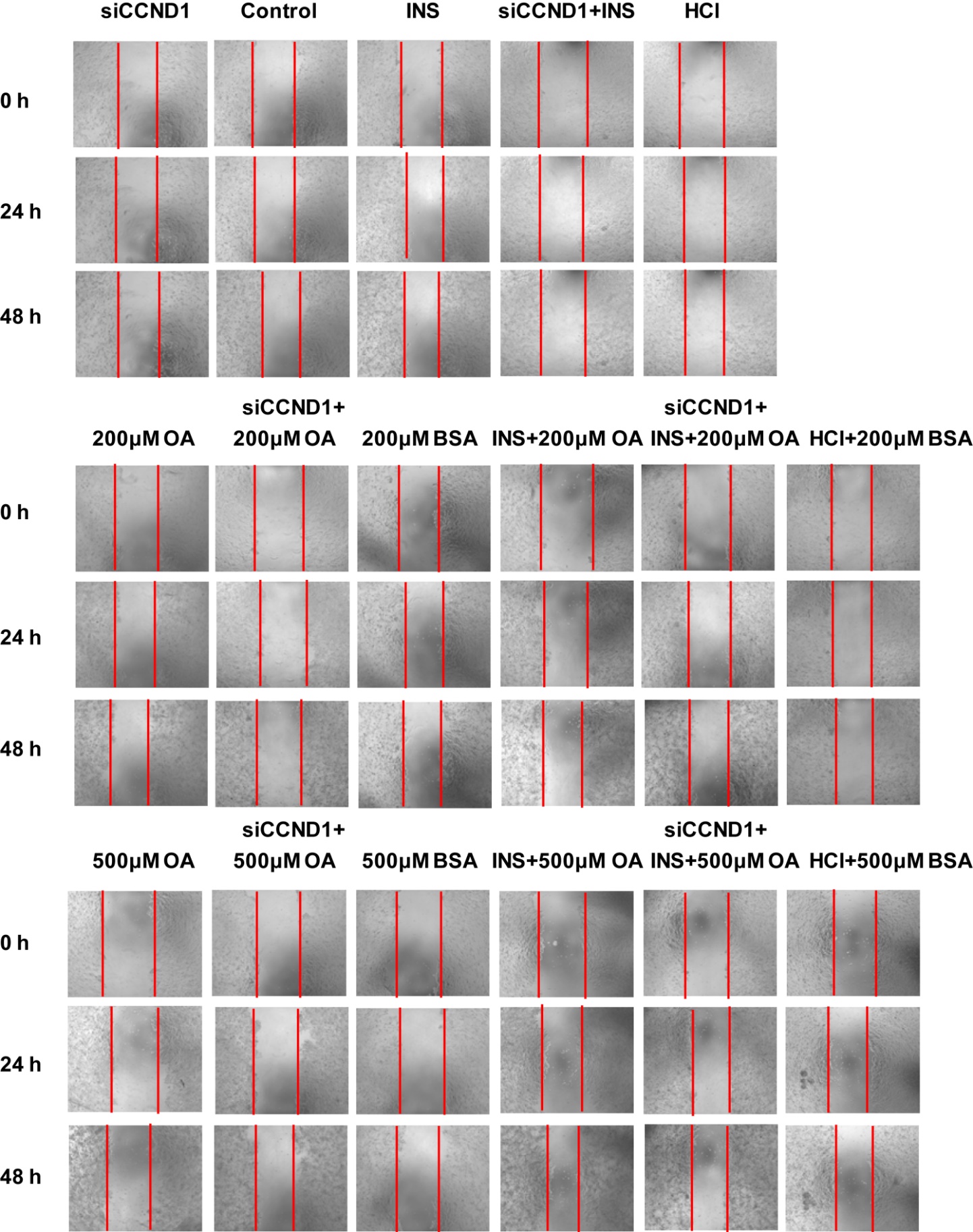


**Figure S6.**  Microscopy image of the wound healing assay on HCT116 cells treated with insulin (50 nM) and/or OA (200 or 500 μM) stimulation with or without cyclin D1 knockdown.


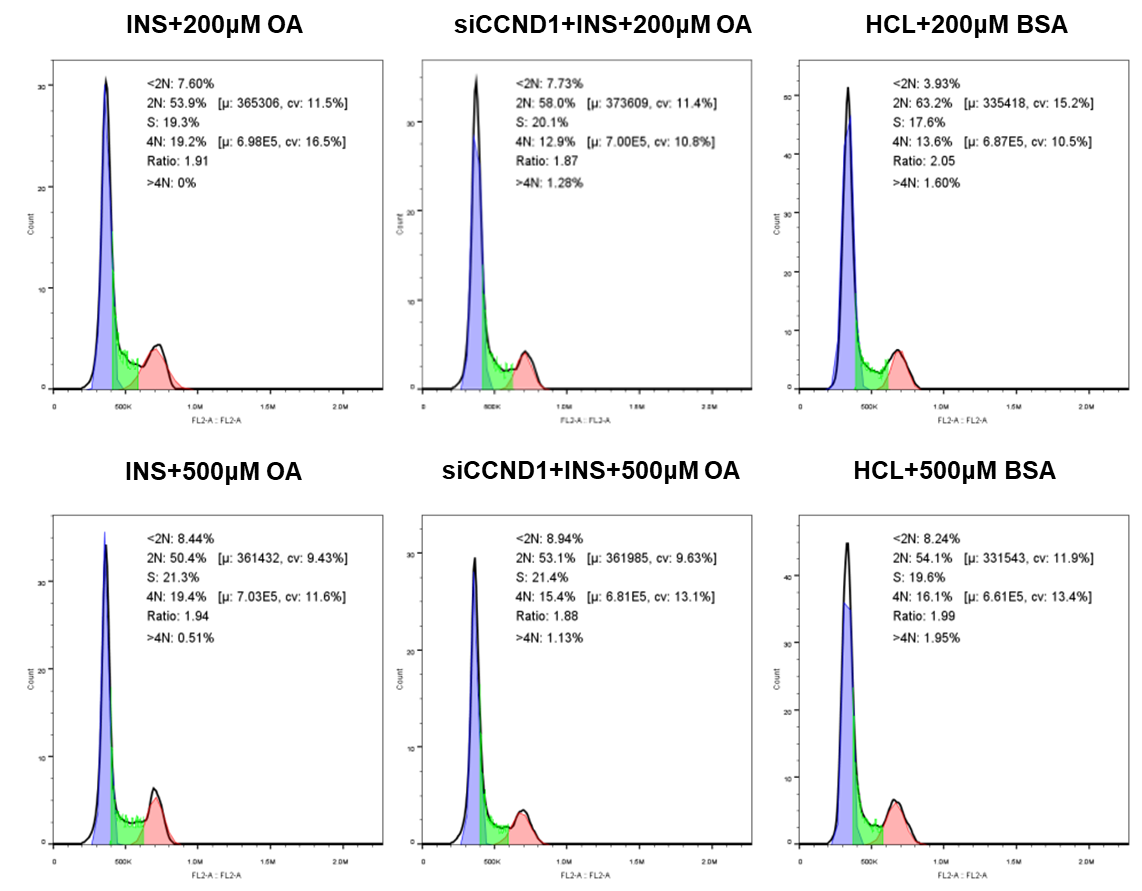


**Figure S7.**  Cell cycle measurement with or without cyclin D1 knockdown using siRNA.


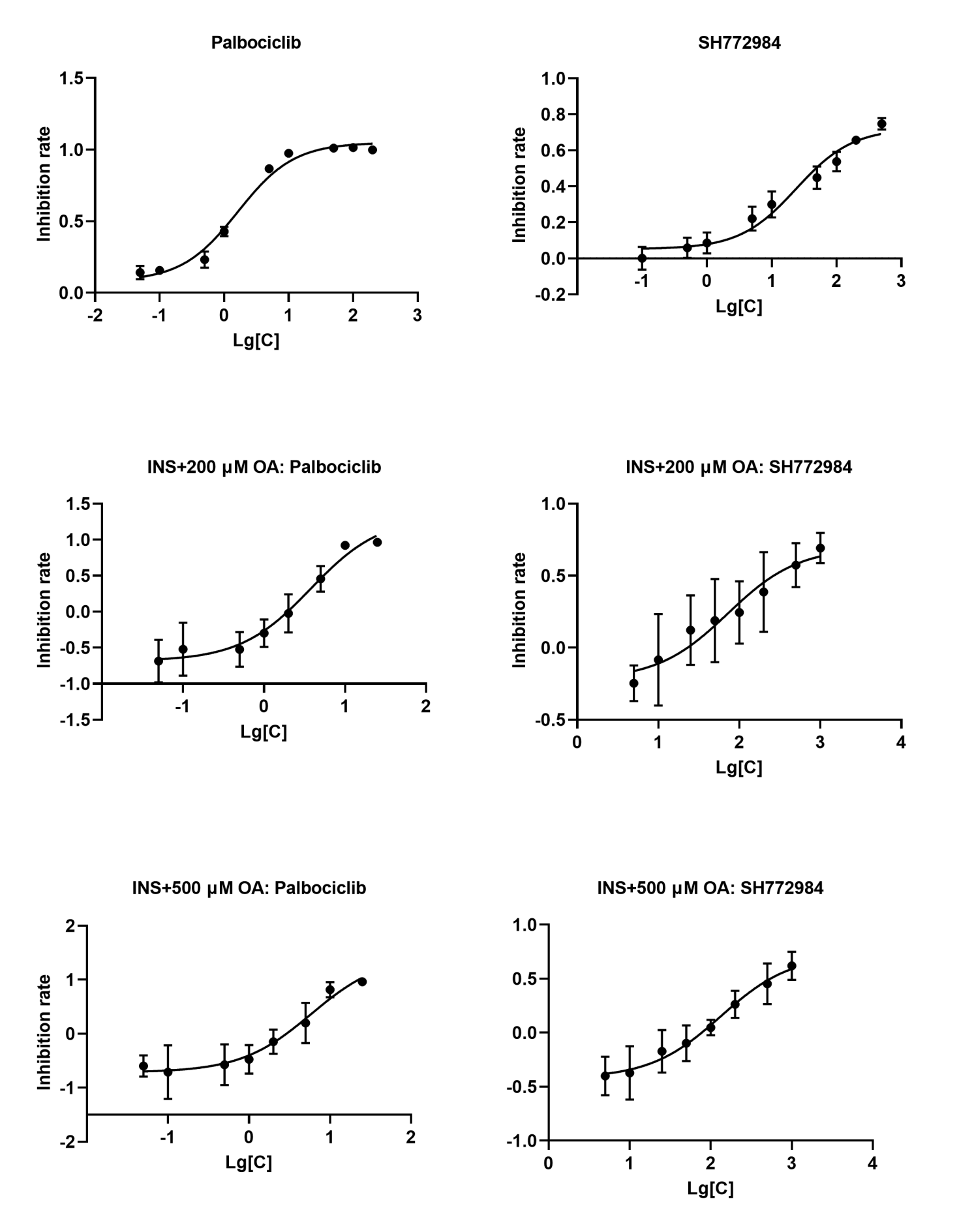


**Figure S8.**  IC_50_ of palbociclib and SCH772984 on HCT116 cell line in different conditions.

**Figure S9.**  Schematic of the *in vivo* experiment performed on CRC xenograft mice.

## Supplementary Tables

Table S1 Summary of existing metabolomics papers related to OA dysregulation in T2D or CRC

| **Disease** | **Patient^*^** | **Healthy control^*^** | **Change trend^$^** | **P value** | **Sample type** | **Analytical platform** | **Reference** |
| --- | --- | --- | --- | --- | --- | --- | --- |
| T2D | 197 | 197 | up-regulated | <0.001 | Serum | LC-MS/GC-MS | [1] |
| T2D | 26 | 26 | up-regulated | 2.36E-01 | Plasma | GC-MS | [2] |
| T2D | 37 | 32 | up-regulated | 5.10E-02 | Plasma | LC-MS/GC-MS | [3] |
| CRC | 64 | 65 | up-regulated | 4.00E-02 | Serum | GC-MS | [4] |
| CRC | 62 | 62 | up-regulated | 9.31E-06 | Serum | LC-MS/GC-MS | [5] |

Note: ^*^ number of cases in the group; ^$^ compared to healthy controls.

References:

[1] Lu Y, Wang Y, Ong CN, Subramaniam T, Choi HW, Yuan JM, Koh WP, Pan A. Metabolic signatures and risk of type 2 diabetes in a Chinese population: an untargeted metabolomics study using both LC-MS and GC-MS. Diabetologia. 2016, 59(11):2349-2359.

[2] Zeng M, Liang Y, Li H, Wang B, Chen X. A metabolic profiling strategy for biomarker screening by GC-MS combined with multivariate resolution method and Monte Carlo. Anal Methods. 2011, 3(2):438-445.

[3] Abu Bakar MH, Sarmidi MR. Association of cultured myotubes and fasting plasma metabolite profiles with mitochondrial dysfunction in type 2 diabetes subjects. Mol Biosyst. 2017, 13(9):1838-1853.

[4] Qiu Y, Cai G, Su M, Chen T, Zheng X, Xu Y, Ni Y, Zhao A, Xu LX, Cai S, Jia W. Serum metabolite profiling of human colorectal cancer using GC-TOFMS and UPLC-QTOFMS. J Proteome Res. 2009, 8(10):4844-50.

[5] Tan B, Qiu Y, Zou X, Chen T, Xie G, Cheng Y, Dong T, Zhao L, Feng B, Hu X, Xu LX, Zhao A, Zhang M, Cai G, Cai S, Zhou Z, Zheng M, Zhang Y, Jia W. Metabonomics identifies serum metabolite markers of colorectal cancer. J Proteome Res. 2013, 7;12(6):3000-9.
